# Contemporary English Pain Descriptors as Detected on Social Media Using Artificial Intelligence and Emotion Analytics Algorithms: Cross-sectional Study

**DOI:** 10.2196/31366

**Published:** 2021-11-25

**Authors:** Ming Yi Tan, Charlene Enhui Goh, Hee Hon Tan

**Affiliations:** 1 Faculty of Dentistry, National University of Singapore Singapore Singapore

**Keywords:** pain descriptors, social media, artificial intelligence, emotion analytics, McGill Pain Questionnaire

## Abstract

**Background:**

Pain description is fundamental to health care. The McGill Pain Questionnaire (MPQ) has been validated as a tool for the multidimensional measurement of pain; however, its use relies heavily on language proficiency. Although the MPQ has remained unchanged since its inception, the English language has evolved significantly since then. The advent of the internet and social media has allowed for the generation of a staggering amount of publicly available data, allowing linguistic analysis at a scale never seen before.

**Objective:**

The aim of this study is to use social media data to examine the relevance of pain descriptors from the existing MPQ, identify novel contemporary English descriptors for pain among users of social media, and suggest a modification for a new MPQ for future validation and testing.

**Methods:**

All posts from social media platforms from January 1, 2019, to December 31, 2019, were extracted. Artificial intelligence and emotion analytics algorithms (Crystalace and CrystalFeel) were used to measure the emotional properties of the text, including *sarcasm*, *anger*, *fear*, *sadness*, *joy*, and *valence*. Word2Vec was used to identify new pain descriptors associated with the original descriptors from the MPQ. Analysis of count and pain intensity formed the basis for proposing new pain descriptors and determining the order of pain descriptors within each subclass.

**Results:**

A total of 118 new associated words were found via Word2Vec. Of these 118 words, 49 (41.5%) words had a count of at least 110, which corresponded to the count of the bottom 10% (8/78) of the original MPQ pain descriptors. The count and intensity of pain descriptors were used to formulate the inclusion criteria for a new pain questionnaire. For the suggested new pain questionnaire, 11 existing pain descriptors were removed, 13 new descriptors were added to existing subclasses, and a new *Psychological* subclass comprising 9 descriptors was added.

**Conclusions:**

This study presents a novel methodology using social media data to identify new pain descriptors and can be repeated at regular intervals to ensure the relevance of pain questionnaires. The original MPQ contains several potentially outdated pain descriptors and is inadequate for reporting the psychological aspects of pain. Further research is needed to examine the reliability and validity of the revised MPQ.

## Introduction

Pain is “an unpleasant sensory and emotional experience associated with, or resembling that associated with, actual or potential tissue damage” [1]. Although the experience of pain is common, each person’s pain is unique and felt physically only by that person. Pain measurement is essential for diagnosis, monitoring of disease progression, and evaluation of treatment effectiveness [2]. This understanding and measurement of pain occur largely through verbal reporting by the person experiencing the pain. Health care providers use this information to characterize and determine pain intensity, and in many instances, make judgments regarding treatment [3]. As verbalization of pain can sometimes be difficult, comprehensive questionnaires to elicit better descriptions of pain for diagnostic accuracy are important.

Various instruments have been developed for the assessment of pain. For acute pain, pain scales that focus on identifying pain location and intensity, such as the visual analog scale and numeric rating scale, are most commonly used [4]. Although shown to be inferior to both the visual analog scale and numeric rating scale, 4-point or 6-point verbal categorical rating scales using adjectives to describe different levels of pain have also been successfully used [5]. For children, pain scales that use images of happy and unhappy faces have been found to be appropriate [6]. All these instruments have been validated in clinical and research settings; however, their accuracy is dependent on timely recording.

Assessment of chronic pain is indisputably more complex. The long-term burden of pain plays a profound role in shaping an individual’s physical and psychological state. In addition, the negative downstream effects of chronic pain can exacerbate the original pain condition through various pathways that remain poorly understood. Owing to these complexities, the above unidimensional instruments that only describe pain in terms of intensity may be too simplistic for meaningful clinical correlation.

Several authors have emphasized the need to recognize the multidimensional aspects of pain [7-9], and 6 dimensions have previously been described: physiological, sensory, affective, cognitive, behavioral, and sociocultural [7]. Health care professionals have acknowledged the varying qualities of pain and often depend on characteristic descriptions of pain to identify eventual diagnoses.

The McGill Pain Questionnaire (MPQ), created in 1971, is one of the most frequently cited instruments and has been validated for use in asymptomatic, symptomatic, and persistently symptomatic populations [10]. Its role in clinical research has been established, in large part because of the multidimensional nature of this instrument [7,11]. The original MPQ [12,13] comprises 78 pain descriptors broadly divided into 3 major classes: sensory, affective, and evaluative. These pain descriptors are further categorized into 20 subclasses, with the words within the respective subclasses ranked based on pain intensity.

The MPQ can be administered by an interviewer who reads the instructions to the patient and defines any words that are not understood [14]. It may also be self-administered. There is heavy reliance on language proficiency, a lack of which may limit the effectiveness of the instrument. Regional linguistic patterns may also be inadvertently incorporated into practice. In addition, language itself is constantly changing and evolving, with use and meanings constantly being updated.

The MPQ has remained unchanged since its inception, and the impact of modern language use on the relevance of MPQ pain descriptors remains unreported. During this time, the invention of the internet and its exponential penetration have drastically reshaped our world. Social media, which comprises forums, blogs, business networks, social gaming, microblogs, photo-sharing platforms, and chat apps, has evolved dramatically alongside these developments. Furthermore, >50% of the global population was expected to access the internet in 2019, and the same figure was expected to use social media platforms [15]. This generates a staggering amount of publicly available social media data, allowing linguistic analysis at a scale that has never been seen before.

Natural language processing or computational linguistics, together with machine learning algorithms, have evolved substantially over the years to be able to analyze, learn, and understand the linguistic contexts of words, identify sentiments and emotions, and form neural network models [16]. These have enabled health researchers to use social media data to address a wide range of public health concerns, including surveillance and inference of developing trends in infectious diseases, targeting of public health strategies, evaluation of public health interventions, ascertaining public perceptions of nonmedical use of opioids, and even predicting disease status [17-21].

In relation to chronic pain, social media represents a snapshot of natural day-to-day colloquial language rather than formal communication. Furthermore, the nature of social media encourages users to capture their thoughts and ideas instantaneously. This is particularly important for accurate pain reporting, which is vulnerable to recall bias and relies heavily on timely reporting. Previous studies have observed extensive amounts of web-based conversations regarding pain [22], suggesting that social media may be a valid place to obtain a large amount of data regarding pain experience. Another study demonstrated that the characteristics of pain conditions could be discerned from social media posts [23], indicating that the depth of data may be sufficient for health care workers to further analyze and understand pain. Therefore, the objectives of this investigation are to use social media data to examine the relevance of pain descriptors from the existing MPQ, identify novel contemporary English descriptors for pain among users of social media, and suggest a modification for a new MPQ for future validation and testing.

## Methods

### Overview

**Figure 1:**
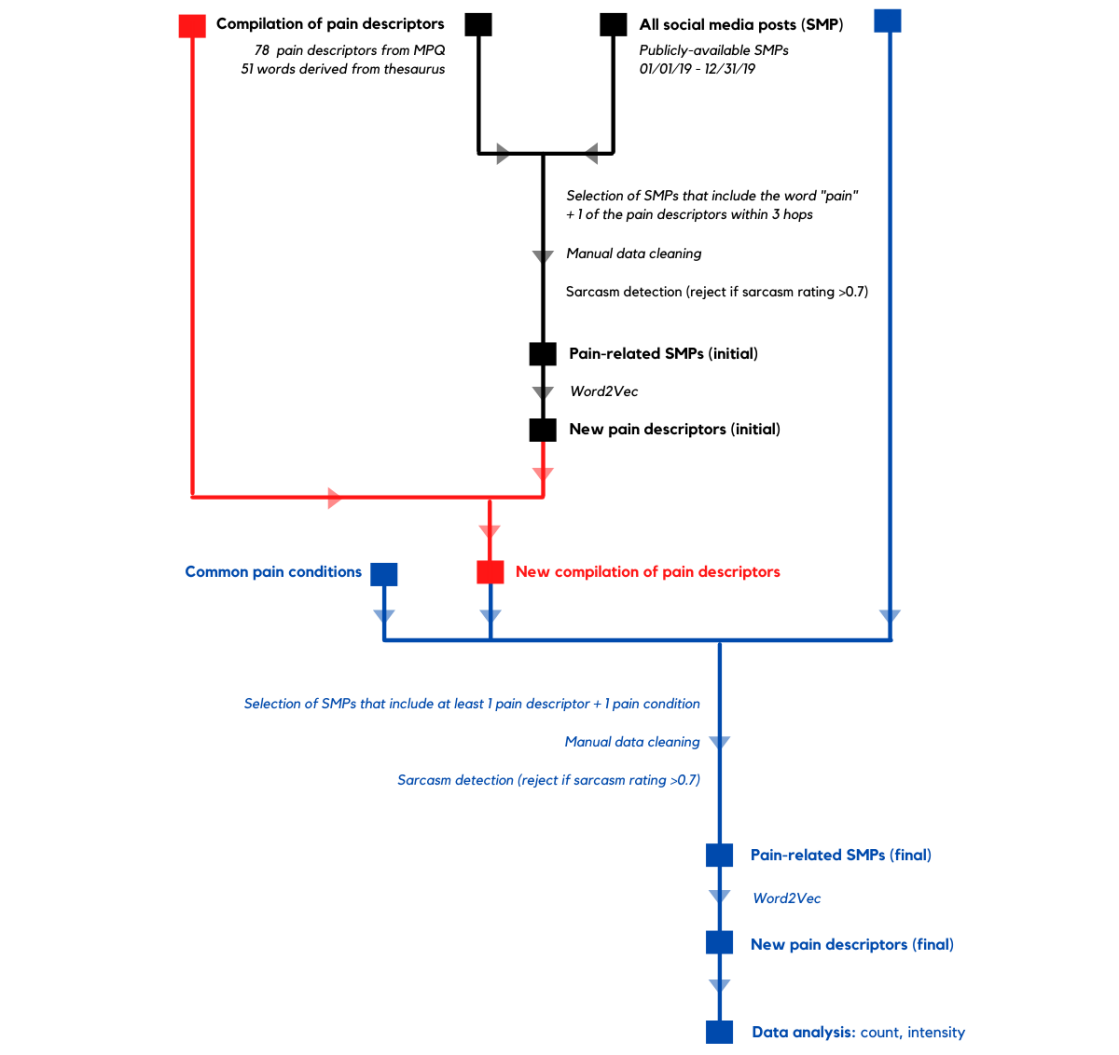
Workflow for the derivation of new pain descriptors. SMP: social media post.

This study used artificial intelligence and emotion analytics algorithms for the derivation and analysis of pain expression from social media platforms. The workflow was executed by a company specializing in linguistic and emotion analytics (INTNT.AI) and comprised 5 main steps conducted in an iterative process: (1) preliminary data gathering, (2) data cleaning, (3) Word2Vec (patent number US9037464B1; Google Inc), (4) final data gathering and cleaning, and (5) data analysis. The workflow is summarized in Figure 1. Preliminary data gathering was first performed to identify new pain descriptors through Word2Vec, which could then be used to maximize the search for relevant social media posts in the final data gathering.

All posts from social media platforms over a 1-year period, from January 1, 2019, to December 31, 2019, were extracted. These data were acquired from a social listening platform (Meltwater) that aggregates and gives direct and official access to all accounts open to the public on Twitter, Facebook, Instagram, and YouTube.

### Preliminary Data Gathering

A list comprising 78 pain descriptors from the MPQ and 51 additions yielded through the use of a mixture of web-based thesauruses was used to identify relevant social media posts (Multimedia Appendix 1). Word networks were created from social media posts using word graphs and path search algorithms that allow the linking of words to the root word of interest [24,25]. Words immediately next to the root word are described as one *hop* away, whereas subsequent layers of words are in increasing levels of hops.

Only social media posts containing the word *pain* and one of the targeted pain descriptors within 3 *hops* were included in this study. The criterion of within 3 *hops* was decided based on pilot testing that showed good richness of social media posts that balanced relevance with the breadth of content.

### Data Cleaning of the Social Media Posts

The selected social media posts were manually scrutinized and cleaned. Usernames, hyperlinks, and internet-specific symbols were removed. In addition, the content of the social media posts was evaluated for relevance. Social media posts that contained the pain descriptor in irrelevant contexts were removed.

The remaining posts were then input into a sarcasm detection machine (Crystalace, Institute of High Performance Computing, Agency for Science, Technology and Research), which is a support vector machine classifier trained with an affect-cognition-sociolinguistics feature model [26,27]. The Crystalace sarcasm detection method rated social media posts for sarcasm on a scale of 0-1, with 1 indicating maximum sarcasm. Social media posts with a sarcasm rating >0.7 were removed from the database.

Sarcasm is a difficult concept to handle in emotion analysis. Traditionally, sarcasm has been viewed from a psychological perspective where overt irony is actively pursued by the speaker as a tool of *verbal violence* [28]. To achieve the required effect and aggression, sarcasm requires a higher level of semantic complexity than normal conversation, in which both positive and negative connotations are interwoven. The CrystalNest software (CrystalNest, Institute of High Performance Computing, Agency for Science, Technology and Research) combines feature engineering and deep learning frameworks with linguistic rules overlay. This performed very well in sarcasm detection for SEMEVAL 2017 and 2018 and was therefore selected for this study [24,25]. Further testing of later models of the CrystalFeel emotion intensity analysis engine also indicated accuracy levels of 0.818, 0.765, 0.765, 0.788, and 0.856 for predicting the intensities of anger, fear, sadness, joy, and valence, respectively [29].

### Word2Vec to Identify Associated Words

**Figure 2:**
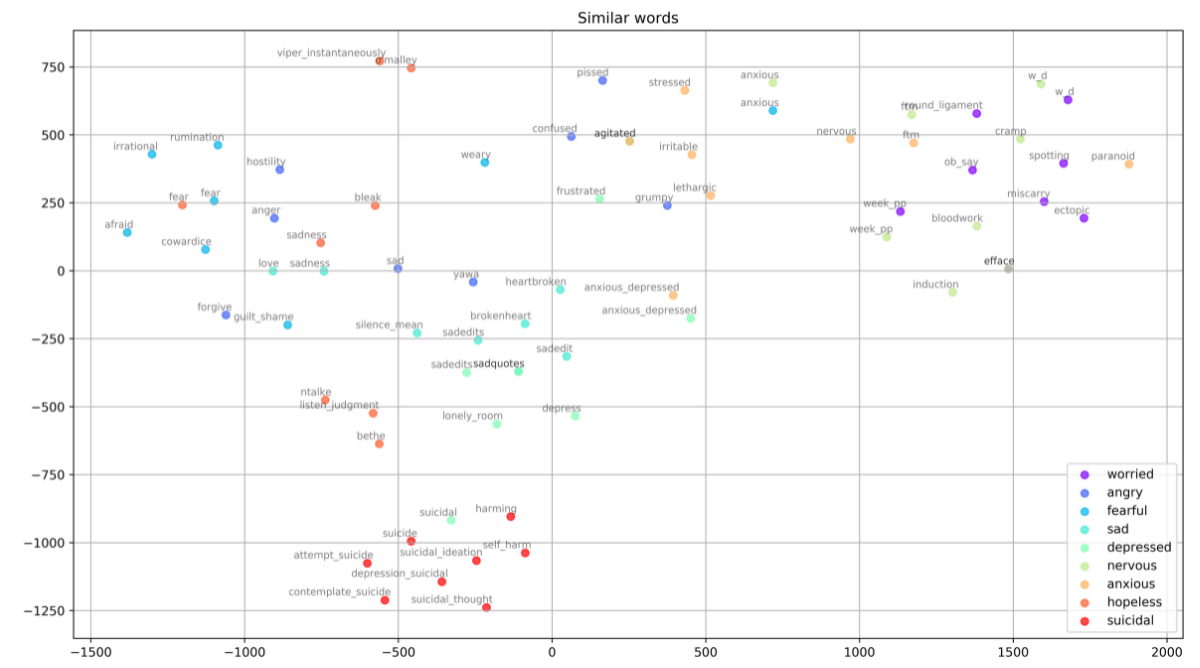
Sample 2D illustration of words with common contexts within the overall 3D vector space; similar words are color-coded. This graph was constructed using T-distributed Stochastic Neighbor Embedding (TSNE), to aid visualization of word clusters. TSNE works by taking a group of high-dimensional vocabulary word feature vectors, then compressing them down to 2-dimensional x, y coordinate pairs. This method keeps similar words close together on the plane, while maximizing the distance between dissimilar words.

Word2Vec [30] was then used on the remaining social media posts to convert the semantic meaning of words into a numerical representation, or vectors, based on the context in which those words occur. Words that share common contexts are located close to one another within the overall vector space constructed from the inputted text (Figure 2).

This process allowed for the classification of pain dimensions from the open text found in the included social media posts and the identification of new pain descriptors. All new words with a positive vector distance from the original list of pain descriptors were considered to be associated, and a maximum of 20 associated words per root word was selected for inclusion.

### Final Data Gathering and Cleaning

Newly identified pain descriptors derived from Word2Vec mechanisms were compiled with the original list of pain descriptors used in preliminary data gathering to form an expanded list of keywords to be used for the final round of data gathering. The search for relevant social media posts was performed in the same social media platforms and period as above.

For greater specificity to health conditions, social media posts were included only if they contained at least one of the pain descriptors in this new list, as well as one pain condition from a list of common pain conditions (Multimedia Appendix 2). This list of pain conditions was not input in the preliminary round of data gathering to ensure maximal identification of possible pain descriptors, regardless of context. The combination of pain descriptors with pain conditions eliminated most of the social media posts that were irrelevant to the study.

The final data set was obtained after the selected social media posts were put through the same data cleaning steps as detailed above.

### Data Analysis

The original MPQ comprised 78 pain descriptors categorized into 20 subclasses. In addition, 51 additional words were yielded through the use of web-based thesauruses. These 129 original pain descriptors served as keywords for the final analysis. The final data set was input into Word2Vec, and the final classification of descriptors was obtained using the algorithm.

Words found to be related to keywords were identically color-coded, located in close proximity to the overall vector space, and had a positive vector distance. A maximum of 20 words found to be most similar to each of the predetermined 129 keywords was selected for inclusion and further pruning. These were evaluated for relevance to pain descriptors. Entries with contrasting meanings to the keywords (eg, *hot* vs *cold*), entries that shared the same root word as the MPQ keywords, and entries irrelevant to pain description were removed.

The number of mentions, or count, of each pain descriptor within the final data set of selected social media posts was computed. Analysis of the counts for the 78 original MPQ keywords was conducted to determine the minimum threshold level for the inclusion of new associated words. The bottom 10% of the original MPQ pain descriptors were found to have counts of <110. Therefore, a count of 110 was set as the minimum threshold level for the prevalence of word use, and all words with a count of <110 were removed.

The intensities of all descriptors were also analyzed using natural language processing emotion analytics algorithms (CrystalFeel, Institute of High Performance Computing, Agency for Science, Technology and Research), which considered the entire sentence or paragraph in which each descriptor was found. The CrystalFeel algorithms allowed for the measurement of the emotional properties in text, including *anger*, *fear*, *sadness*, *joy*, and *valence*. Intensity was reflected on a scale of 0-1, with 1 implying maximum pain intensity. This formed the basis for determining the order of pain descriptors within each subclass.

## Results

### Sample of Social Media Posts

A total of 572,742 social media posts were obtained from a preliminary round of data gathering. Following manual evaluation of the 572,742 social media posts for relevance, 8310 (1.45%) social media posts were removed, whereas 7824 (1.37%) social media posts were removed after failing to meet the threshold criterion for sarcasm. Word2Vec identified 34 new pain descriptors that were used together with the original list of words to widen the search for relevant social media posts in the second round of data gathering. A total of 1,877,122 social media posts were identified in the second round of data gathering. After data cleaning and the additional inclusion criteria of containing at least one pain condition as well as one pain descriptor in the social media post, 11.55% (216,873/1,877,122) of social media posts remained for the final data analysis.

### Identification of New Pain Descriptors Through Word2Vec

**Figure 3:**
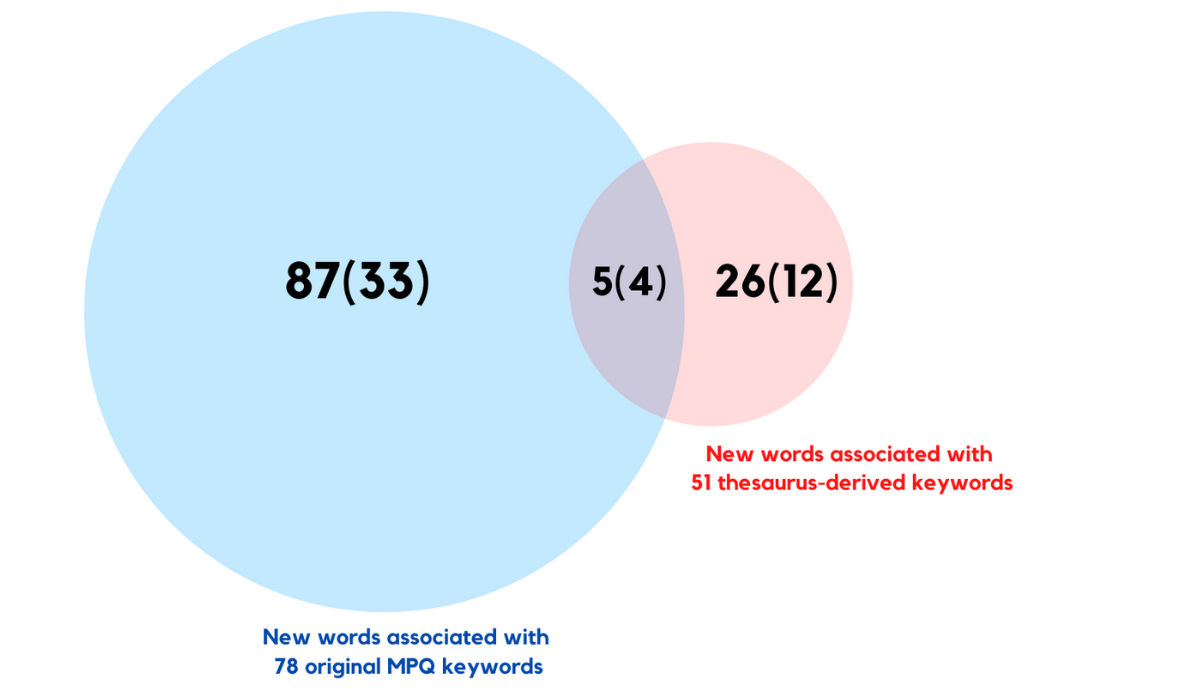
A total of 118 new words were found to be associated with the 78 original McGill Pain Questionnaire keywords and 51 thesaurus-derived keywords; bracketed values indicate the number of words with count ≥110. MPQ: McGill Pain Questionnaire.

Using Word2Vec, a total of 118 new associated words were found for the 129 pain descriptor keywords defined in this study, following the removal of repetitions. Of these 118 words, 5 (4.2%) were associated with both the original MPQ and thesaurus-derived keywords, 87 (73.7%) were associated with MPQ keywords only, and 26 (22%) were associated with thesaurus-derived keywords only (Figure 3).

### Count and Intensity of the Original MPQ Keywords and New Pain Descriptors

Of the 118 new associated words acquired through Word2Vec, 49 (41.5%) words were found to have a count of at least 110, meeting the minimum threshold level for the prevalence of word use.

**Figure 4:**
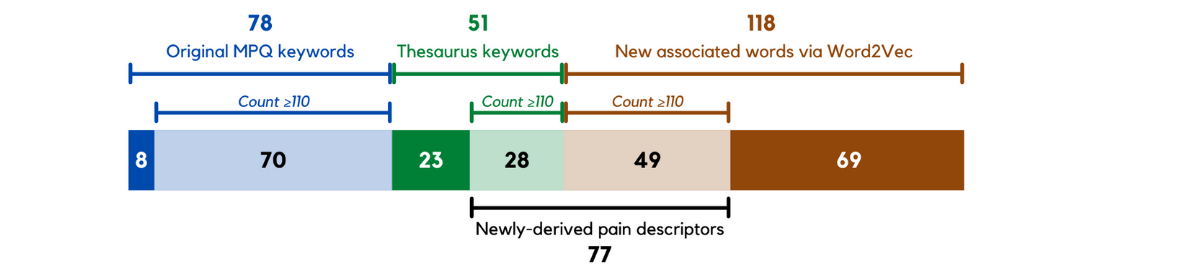
Breakdown of original and newly derived pain descriptors. MPQ: McGill Pain Questionnaire.

28 (23.7%) thesaurus-derived keywords met the minimum threshold count of 110. These and the 49 out of 118 (41.5%) new associated words derived through Word2Vec were combined into a single list for further evaluation, representing the final list of 77 newly derived pain descriptors (Figure 4). From this list, the pain descriptors that received the top 10 highest counts in descending order were *anxiety* (96909), *depression* (65223), *fear* (48165), *excruciating* (32094), *anger* (31506), *discomfort* (31333), *depress* (29822), *sadness* (25817), *low* (23027), and *fever* (21441).

Of the 78 original MPQ keywords, the pain descriptors that received the top 10 highest counts in descending order were *sharp* (26679), *hot* (25405), *tingling* (25405), *intense* (24269), *numb* (23964), *unbearable* (20929), *cold* (19099), *sore* (16127), *burning* (14204), and *itchy* (14204).

The intensity of the 78 original MPQ keywords ranged from 0.367 (for *flickering* and *jumping*) to 0.699 (for *terrifying*). For the combined list of 77 newly derived pain descriptors, the intensity ranged from 0.325 (for *scratching*) to 0.7467 (for *suicidal*).

The counts and intensities of existing and new words are presented in Multimedia Appendix 3.

### Suggested New Pain Questionnaire

#### Overview

Analyses of count and intensity of the pain descriptors, as well as manual analysis of the social media posts for the context in which the word was used, were used to recommend the inclusion of pain descriptors in a new pain questionnaire. The suggested changes, categorized by the MPQ subclasses, are summarized in Table 1.

**Table 1:** Comparison of the original McGill Pain Questionnaire (MPQ) with the suggestion for a new pain questionnaire.

Subclass and pain descriptors from the original MPQ			Suggested words for a new pain questionnaire		Ranking reordered? (yes or no)
**Temporal**					
	Flickering Quivering (removed) Pulsing Throbbing Beating (removed) Pounding	Flickering Pulsing Throbbing Pounding		No	
**Spatial**					
	Jumping Flashing Shooting	Jumping Flashing Shooting		No	
**Punctuate pressure**					
	Pricking Boring Drilling Stabbing Lancinating (removed)	Pricking Boring Drilling Stabbing Puncturing (new)		No	
**Incisive pressure**					
	Sharp Cutting Lacerating (removed)	Cutting Sharp		Yes	
**Constrictive pressure**					
	Pinching Pressing Gnawing Cramping Crushing	Pressing Gnawing Crushing Pinching Cramping		Yes	
**Traction pressure**					
	Tugging Pulling Wrenching	Tugging Contraction (new) Pulling Wrenching Clenching (new)		No	
**Thermal**					
	Hot Burning Scalding (removed) Searing	Hot Searing Burning		Yes	
**Brightness**					
	Tingling Itchy Smarting (removed) Stinging	Scratching (new) Tingling Itchy Stinging		No	
**Dullness**					
	Dull Sore Hurting Aching Heavy	Sore Aching Dull Hurting Heavy		Yes	
**Sensory miscellaneous**					
	Tender Taut (removed) Rasping (removed) Splitting	Tender Splitting		No	
**Tension**					
	Tiring Exhausting	Tiring Straining (new) Exhausting		No	
**Autonomic**					
	Sickening Suffocating	Sickening Suffocating		No	
**Fear**					
	Fearful Frightful (removed)	Fearful Horrendous (new) Horrifying (new)		No	
**Punishment**					
	Terrifying Punishing (removed) Grueling Cruel Vicious Killing	Grueling Cruel Vicious Killing Terrifying		Yes	
**Affective-evaluative-sensory miscellaneous**					
	Wretched Blinding	Wretched Blinding		No	
**Evaluative**					
	Annoying Troublesome Miserable Intense Unbearable	Mild (new) Troublesome Intense Annoying Irritating (new) Unbearable Horrible (new) Miserable Excruciating (new) Distressing (new)		Yes	
**Supplementary a**					
	Spreading Radiating Penetrating Piercing	Spreading Radiating Penetrating Piercing		No	
**Supplementary b**					
	Tight Numb Drawing (removed) Squeezing Tearing	Bruising (new) Tight Numb Squeezing Tearing		No	
**Supplementary c**					
	Cool Cold Freezing	Cool Cold Freezing		No	
**Supplementary d**					
	Nagging Nauseating Agonizing Dreadful Torturing	Nagging Nauseating Agonizing Dreadful Torturing		No	
**Psychological (new)**					
	—^a^	Worried (new) Angry (new) Fearful Sad (new) Depressed (new) Nervous (new) Anxious (new) Feel hopeless (new) Suicidal (new)		N/A^b^	

^a^Not available.

^b^N/A: not applicable.

#### Removal of Words

Of the 78 pain descriptors, 8 (10%) pain descriptors from the original MPQ were removed because of low use (count<110). The words removed were *quivering* (from the *Temporal* subclass), *lancinating* (from the *Punctuate Pressure* subclass), *lacerating* (from the *Incisive Pressure* subclass), *scalding* (from the *Thermal* subclass), *smarting* (from the *Brightness* subclass), *taut* and *rasping* (from the *Sensory miscellaneous* subclass), and *frightful* (from the *Fear* subclass).

In addition, of the 78 pain descriptors from the original MPQ, 3 (4%) pain descriptors were removed because, on manual analysis of the social media posts, the contexts in which they were used were found to have deviated from pain description. The word *beating* (from the *Temporal* subclass) was mainly used as a synonym for *overcoming pain* or the physical action of a beating instead of being used as a pain descriptor. *Punishing* (from the *Punishment* subclass) and *drawing* (from the *Supplementary b* subclass) were also removed as these words were more often used as verbs rather than adjectives.

#### Addition of Words

A total of 13 new associated words were added to the pre-existing subclasses. The added words were *puncturing* (to the *Punctuate Pressure* subclass); *contraction* and *clenching* (to the *Traction Pressure* subclass); *scratching* (to the *Brightness* subclass); *straining* (to the *Tension* subclass); *horrendous* and *horrifying* (to the *Fear* subclass); *mild*, *irritating*, *horrible*, *excruciating*, and *distressing* (to the *Evaluative* subclass); and *bruising* (to the *Supplementary b* subclass).

#### Creation of a Psychological Subclass

An entirely new *Psychological* subclass was suggested to acknowledge the nonphysical aspects of pain. This subclass comprised 9 newly identified descriptors relevant to the description of the emotional state of a person. These were selected for their high counts, which ranged from 1139 to 65,223. Intensity was also taken into consideration during this selection to ensure good graduality of pain description, and this ranged from 0.47 to 0.7467.

The identified pain descriptors consisted of a mixture of nouns and adjectives. For consistency, the 9 selected descriptors were modified to fit into the sentence *“*My pain makes me ___*.*” This resulted in the final selection of *worried*, *angry*, *fearful*, *sad*, *depressed*, *nervous*, *anxious*, *feel hopeless*, and *suicidal* for this new subclass.

#### Reordering of Pain Descriptors to Reflect Pain Intensity

The pain descriptors of 6 of the 20 subclasses were reordered based on their emotional intensity to reflect decreasing pain intensity. These new rankings are shown in Table 1.

## Discussion

### Principal Findings

This study provides insights into modern language use in the context of pain description. We found infrequent use and even a change in context for the use of several descriptors from the original MPQ, reflecting the evolution of language and suggesting limitations in the current MPQ. We also identified several new pain descriptors that can be used to update the MPQ, including the emergence of a possible *Psychological* subclass of pain descriptors that can capture the emotional and mental aspects of pain. These changes may improve the application of the MPQ in physical settings and digital telehealth platforms. An updated MPQ with relevant and appropriate pain descriptors may improve the ability of the MPQ to accurately characterize the pain experience, leading to more tailored diagnoses and treatment plans. In addition, better awareness of modern language use can also influence the design of conversational artificial intelligence systems, commonly known as *chatbots*, which have the potential to decrease the need for physical encounters in health care facilities [31]. The linguistic styles of chatbots are thought to affect relationship building between patients and the respective chatbots [32].

The top 10 highest counts for the 78 original MPQ keywords were substantially lower than those for the new words, ranging from 14,204 to 26,679 and from 21,441 to 96,909, respectively. This suggests that words previously selected for the MPQ may no longer be as commonly used today.

Interestingly, 6 out of the top 10 newly identified pain descriptors that received the top 10 highest counts were relevant to the emotional or mental description of pain, namely *anxiety*, *depression*, *fear*, *anger*, *depress*, and *sadness*, which led to the emergence of a *Psychological* subclass.

Our study combined the big data afforded by social media posts with artificial intelligence and additional emotion analytics algorithms that allow for a broad analysis of social media posts with high speed and accuracy. The combination of pain research with this technology, originally designed to help businesses understand what their customers want through linguistic and contextual cues, helps to address a pressing health care need. As much as 40% of the population contends with chronic pain, and the extensive cumulative impact of chronic pain in the United States alone was estimated to exceed US $500 billion annually [33].

The updated pain definition by the International Association for the Study of Pain highlights the subjective and emotional nature of pain. The literature reports various links between pain and mood or psychiatric disorders. For instance, pain and major depressive disorder often occur concurrently, appearing to mutually exacerbate the severity of the individual conditions [34-37]. Chronic pain has been found to increase the risk of depressive disorders and comorbidity; inversely, depression is also a risk factor for the later development of chronic pain [38-42]. Although not fully understood, several studies have reported shared biological pathways and neurotransmitters between both conditions [43-45]. The involvement of the limbic structures of the brain in both pain processing and depressed mood can be triggered by stress and indicates the sharing of neurobiological factors between pain and depression [46-49]. In addition, the pain experienced may be altered by emotions such as anger expression [50], anxiety, or the feeling of powerlessness, with 1 author suggesting that reducing the perception of powerlessness may reduce pain intensity [51].

Unfortunately, these psychological, emotional, and behavioral interactions with pain experience are rarely brought up in patient interviews. The existing MPQ focuses largely on the physical description of pain. Although its existing words allow the interviewer to infer the emotional toll of pain on the patient, there is lack of a dedicated segment that acknowledges the psychological burden of pain. The introduction of a new *Psychological* subclass is a significant change from the existing MPQ, prompting patients to definitively select the resultant emotion caused by their pain. The patient’s choice from the selection of *worried*, *angry*, *fearful*, *sad*, *depressed*, *nervous*, *anxious*, *feel hopeless*, and *suicidal* may also indicate the patient’s primary coping mechanism in the face of the challenges of the pain condition. The behavior of a patient who feels *anger* may be starkly different from another who feels *fearful* and could aid future interventions or referrals by health care providers.

### Limitations and Future Direction

This study has some limitations. Only social media posts in English were included in this study, and most social media posts originated from the Western hemisphere. Therefore, the application of these findings is largely limited to the United States and may not be generalizable to other English-speaking countries. Future work using similar artificial intelligence mechanisms stratified by geographical regions to address regional linguistic differences, and perhaps different languages, may be explored.

Similarly, the sample population in this study was limited to users of social media platforms. The preferred social media platform differs according to age, and in general, the overall population tends to skew young [52,53]. It is possible that the pain descriptors found are specific to this population only; therefore, the results may not be generalizable to older adults for whom the older MPQ terminologies may still apply. There may also be a lack of representation of people of lower socioeconomic status or those whose pain conditions severely compromise daily living [54].

Although the artificial intelligence and emotion analytics algorithms selected for the study had previously demonstrated good accuracy, we did not evaluate the validity of the newly identified pain descriptors. The complexity of human thought and expression makes further validation of the suggested questionnaire on a sample of the target patients necessary. Future research could involve a qualitative focus group to examine the face validity of the word descriptors and to ensure that patients feel that their pain is adequately described by the options available or to suggest other terms. Validation and reliability of the new pain questionnaire should be conducted, and the proposed reordering of words within each subclass should be tested.

### Conclusions

The original MPQ is inadequate for reporting the psychological aspects of pain. Several descriptors from the original MPQ were also noted to have infrequent use or changes in context. This study used artificial intelligence and emotion analytics algorithms to identify contemporary vocabulary for pain description. The described methodology could be repeated at regular intervals to ensure the relevance of the pain questionnaires. Further research is needed to examine the reliability and validity of the revised MPQ.

## Appendix

Multimedia Appendix 1 Pain descriptors for the first round of data gathering.

Multimedia Appendix 2 List of common pain conditions.

Multimedia Appendix 3 Counts and intensity of pain descriptors from the McGill Pain Questionnaire, web-based thesauruses, and identified through Word2Vec.

## Abbreviations

MPQ: McGill Pain Questionnaire
