# Supplementary material for: Contemporary English Pain Descriptors as Detected on Social Media Using Artificial Intelligence and Emotion Analytics Algorithms: Cross-sectional Study
Source: JMIR Form Res. 2021 Nov 25;5(11):e31366. doi: 10.2196/31366 (PMC8663651; doi:10.2196/31366)
Supplement: Multimedia Appendix 1 [file formative_v5i11e31366_app1.docx]

Appendix A. Pain descriptors for first round of data gathering

| **Pain descriptors from McGill Pain Questionnaire**   \| Flickering  Quivering  Pulsing  Throbbing  Beating  Pounding  Jumping  Flashing  Shooting  Pricking  Boring  Drilling  Stabbing  Lancinating  Sharp  Cutting  Lacerating  Pinching  Pressing  Gnawing  Cramping  Crushing  Tugging  Pulling  Wrenching  Hot \| Burning  Scalding  Searing  Tingling  Itchy  Smarting  Stinging  Dull  Sore  Hurting  Aching  Heavy  Tender  Taut  Rasping  Splitting  Tiring  Exhausting  Sickening  Suffocating  Fearful  Frightful  Terrifying  Punishing  Gruelling  Cruel \| Vicious  Killing  Wretched  Blinding  Annoying  Troublesome  Miserable  Intense  Unbearable  Spreading  Radiating  Penetrating  Piercing  Tight  Numb  Drawing  Squeezing  Tearing  Cool  Cold  Freezing  Nagging  Nauseating  Agonizing  Dreadful  Torturing \| \| --- \| --- \| --- \| |
| --- | --- | --- | --- |
| **Pain Descriptors derived through use of thesaurus**   \| Puncture  Slashing  Incision  Jerking  Traction  Hotness  Abrasiveness  Dullness  Mild  Discomfort  Distressing  Horrible  Excruciating  Agony  Fever  Illness  Injury \| Irritation  Misery  Sickness  Spasm  Trouble  Twinge  Wound  Affliction  Catch  Convulsion  Crick  Gripe  Malady  Pang  Stitch  Paroxysm  Throe \| Constant  Nervous  Anxious  On edge  Worry  Feeling down  Depressed  Hopeless  Feeling tired  Trouble concentrating  Anxiety  Depression  Anger  Joy  Fear  Sadness  Shame \| \| --- \| --- \| --- \| |
