# Supplementary material for: Contemporary English Pain Descriptors as Detected on Social Media Using Artificial Intelligence and Emotion Analytics Algorithms: Cross-sectional Study
Source: JMIR Form Res. 2021 Nov 25;5(11):e31366. doi: 10.2196/31366 (PMC8663651; doi:10.2196/31366)
Supplement: Multimedia Appendix 2 [file formative_v5i11e31366_app2.docx]

Appendix B. List of common pain conditions

| Low back pain  Lower back pain  Bad back  Headache  Tension headache  Migraine  Fibromyalgia  Endometriosis  Vulvodynia  Interstitial cystitis  Irritable bowel syndrome  Temporomandibular disorder  Chronic fatigue syndrome  Arthritis  Osteoarthritis  Degenerative joint disease  Rheumatoid arthritis  Vertebrae disc disease  Slipped disc  Myofascial pain  Plantar fasciitis  Muscle pain  Whiplash  Joint pain  Musculoskeletal joint  Gout  Lyme disease  Multiple sclerosis | Shingles  Nerve damage  Neuropathic  Nerve irritation  Diabetic neuropathy  Trigeminal neuralgia  Post-herpetic neuralgia  Carpal tunnel syndrome  Amputee pain  Phantom pain  Temporal arteritis  Giant cell arteritis  Sickle cell disease  Bladder pain syndrome  Painful bladder syndrome  Chronic pelvic pain  Urologic chronic pelvic pain  Radiculopathy  Lumbago  Sciatica  Spondylosis  Otitis media  Maxillary sinusitis  Neck pain  Chest pain  Abdominal pain  Heel pain  Cancer | Toothache  Periodontal disease  Gum disease  Burning mouth syndrome  Cracked tooth  Atypical odontolgia  Chronic idiopathic facial pain  Dental pain  Ear pain  Tinnitus  Medication overuse headache  Myalgia  Head  Neck  Upper limb  Chest  Upper back  Lower back  Abdomen  Groin  Pelvis  Lower limb  Deep  Superficial  Central  Peripheral  Visceral |
| --- | --- | --- |
