# Supplementary material for: Contemporary English Pain Descriptors as Detected on Social Media Using Artificial Intelligence and Emotion Analytics Algorithms: Cross-sectional Study
Source: JMIR Form Res. 2021 Nov 25;5(11):e31366. doi: 10.2196/31366 (PMC8663651; doi:10.2196/31366)
Supplement: Multimedia Appendix 3 [file formative_v5i11e31366_app3.docx]

*Multimedia Appendix 3. Counts and intensity of pain descriptors from the MPQ, online thesauruses, and identified through Word2Vec*

|  | **Keyword** | **Count** | **Intensity** | **Associated Words identified through Word2Vec, if Count >110** | **Count** | **Intensity** |
| --- | --- | --- | --- | --- | --- | --- |
| **Keywords from the original McGill Pain Questionnaire** | | | | | | |
| Temporal | Flickering | 117 | 0.367 |  |  |  |
|  | Quivering | 71 | 0.5881 |  |  |  |
|  | Pulsing | 626 | 0.537 |  |  |  |
|  | Throbbing | 4748 | 0.573 |  |  |  |
|  | Beating | 1890 | 0.5529 |  |  |  |
|  | Pounding | 2152 | 0.596 |  |  |  |
| Spatial | Jumping | 1200 | 0.367 |  |  |  |
|  | Flashing | 431 | 0.537 |  |  |  |
|  | Shooting | 7177 | 0.573 |  |  |  |
| Punctuate Pressure | Pricking | 150 | 0.498 |  |  |  |
|  | Boring | 2000 | 0.402 |  |  |  |
|  | Drilling | 359 | 0.517 |  |  |  |
|  | Stabbing | 6878 | 0.55 | Puncturing | 1529 | 0.6012 |
|  | Lancinating | 23 | 0.5103 |  |  |  |
| Incisive Pressure | Sharp | 26679 | 0.611 |  |  |  |
|  | Cutting | 3883 | 0.485 | Tearing | 1646 | 0.5222 |
|  | Lacerating | 13 | 0.5528 |  |  |  |
| Constrictive Pressure | Pinching | 848 | 0.594 | Squeezing | 2183 | 0.5428 |
|  |  |  |  | Twinge | 214 | 0.5934 |
|  |  |  |  | Low | 23027 | 0.5618 |
|  | Pressing | 2350 | 0.484 |  |  |  |
|  | Gnawing | 502 | 0.549 |  |  |  |
|  | Cramping | 6283 | 0.640 |  |  |  |
|  | Crushing | 2337 | 0.574 |  |  |  |
| Traction Pressure | Tugging | 222 | 0.451 | Pulling | 2664 | 0.492 |
|  | Pulling | 2664 | 0.492 | Pressure | 8161 | 0.6676 |
|  | Wrenching | 1241 | 0.574 | Rend | 547 | 0.5626 |
|  |  |  |  | Sadness | 25817 | 0.515 |
|  |  |  |  | Clenching | 203 | 0.692 |
|  |  |  |  | Contraction | 3356 | 0.427 |
| Thermal | Hot | 25405 | 0.506 | Warm | 1211 | 0.5159 |
|  | Burning | 14204 | 0.537 |  |  |  |
|  | Scalding | 108 | 0.5503 |  |  |  |
|  | Searing | 3110 | 0.531 | Excruciating | 32094 | 0.5961 |
| Brightness | Tingling | 25405 | 0.504 |  |  |  |
|  | Itchy | 14204 | 0.512 | Scratchy | 144 | 0.5335 |
|  |  |  |  | Scratching | 268 | 0.325 |
|  | Smarting | 108 | 0.5435 |  |  |  |
|  | Stinging | 3110 | 0.592 | Tingly | 124 | 0.6132 |
| Dullness | Dull | 8693 | 0.516 |  |  |  |
|  | Sore | 16127 | 0.5 | Swollen | 1077 | 0.6342 |
|  | Hurting | 8569 | 0.551 | Sadness | 25817 | 0.515 |
|  |  |  |  | Hopeless | 2118 | 0.6131 |
|  | Aching | 7735 | 0.506 |  |  |  |
|  | Heavy | 12708 | 0.573 | Mild | 12669 | 0.5141 |
|  |  |  |  | Start | 6996 | 0.5753 |
| Sensory Miscellaneous | Tender | 2924 | 0.5524 |  |  |  |
|  | Taut | 18 | 0.4743 | Stretched | 121 | 0.5198 |
|  | Rasping | 16 | 0.6019 |  |  |  |
|  | Splitting | 1785 | 0.5513 |  |  |  |
| Tension | Tiring | 639 | 0.673 | Stressful | 186 | 0.5755 |
|  |  |  |  | Straining | 405 | 0.5733 |
|  | Exhausting | 2441 | 0.575 | Constant | 8693 | 0.5914 |
| Autonomic | Sickening | 1194 | 0.566 |  |  |  |
|  | Suffocating | 406 | 0.628 |  |  |  |
| Fear | Fearful | 434 | 0.48 | Afraid | 1125 | 0.4335 |
|  |  |  |  | Anxious | 2257 | 0.5854 |
|  | Frightful | 32 | 0.5955 |  |  |  |
|  | Terrifying | 1196 | 0.699 | Horrifying | 285 | 0.6282 |
|  |  |  |  | Excruciating | 32094 | 0.5961 |
|  |  |  |  | Rend | 547 | 0.5626 |
|  |  |  |  | Horrendous | 393 | 0.5401 |
|  |  |  |  | Appalling | 189 | 0.6441 |
| Punishment | Punishing | 1590 | 0.5472 |  |  |  |
|  | Gruelling | 183 | 0.5065 |  |  |  |
|  | Cruel | 7991 | 0.52 |  |  |  |
|  | Vicious | 1552 | 0.54 |  |  |  |
|  | Killing | 13055 | 0.551 |  |  |  |
| Affective-Evaluative-Sensory Miscellaneous | Wretched | 280 | 0.552 | Torment | 290 | 0.5913 |
|  | Blinding | 1018 | 0.522 |  |  |  |
| Evaluative | Annoying | 5402 | 0.528 | Mild | 12669 | 0.5141 |
|  |  |  |  | Constant | 8693 | 0.5914 |
|  |  |  |  | Irritating | 3695 | 0.5291 |
|  | Troublesome | 249 | 0.49 | Discomfort | 31333 | 0.49 |
|  |  |  |  | Mild | 12669 | 0.5141 |
|  | Miserable | 3824 | 0.59 | Horrible | 16042 | 0.5791 |
|  |  |  |  | Constant | 8693 | 0.5914 |
|  |  |  |  | Bad | 14067 | 0.6098 |
|  |  |  |  | Excruciating | 32094 | 0.5961 |
|  |  |  |  | Sick | 5619 | 0.5953 |
|  | Intense | 24269 | 0.518 | Excruciating | 32094 | 0.5961 |
|  |  |  |  | Constant | 8693 | 0.5914 |
|  |  |  |  | Horrible | 16042 | 0.5791 |
|  |  |  |  | Discomfort | 31333 | 0.49 |
|  |  |  |  | Painful | 3717 | 0.5725 |
|  |  |  |  | Severe | 7961 | 0.6242 |
|  | Unbearable | 20929 | 0.546 | Excruciating | 32094 | 0.5961 |
|  |  |  |  | Horrible | 16042 | 0.5791 |
|  |  |  |  | Constant | 8693 | 0.5914 |
|  |  |  |  | Bad | 14067 | 0.6098 |
|  |  |  |  | Hurt | 5086 | 0.6298 |
| Supplementary a | Spreading | 3865 | 0.483 |  |  |  |
|  | Radiating | 3407 | 0.546 |  |  |  |
|  | Penetrating | 226 | 0.562 |  |  |  |
|  | Piercing | 2138 | 0.573 |  |  |  |
| Supplementary b | Tight | 8229 | 0.484 | Stretched | 121 | 0.5198 |
|  | Numb | 23964 | 0.536 | Hurt | 5086 | 0.6298 |
|  |  |  |  | Tingly | 124 | 0.6132 |
|  | Drawing | 1736 | 0.4764 |  |  |  |
|  | Squeezing | 2484 | 0.536 |  |  |  |
|  | Tearing | 1646 | 0.522 | Bruising | 633 | 0.471 |
| Supplementary c | Cool | 6774 | 0.461 |  |  |  |
|  | Cold | 19099 | 0.526 |  |  |  |
|  | Freezing | 808 | 0.591 |  |  |  |
| Supplementary d | Nagging | 1765 | 0.484 | Constant | 8693 | 0.5914 |
|  | Nauseating | 202 | 0.536 | Lightheaded | 282 | 0.6665 |
|  | Agonizing | 5532 | 0.455 | Excruciating | 32094 | 0.5961 |
|  |  |  |  | Horrible | 16042 | 0.5791 |
|  |  |  |  | Constant | 8693 | 0.5914 |
|  | Dreadful | 814 | 0.536 | Excruciating | 32094 | 0.5961 |
|  |  |  |  | Horrible | 16042 | 0.5791 |
|  | Torturing | 846 | 0.522 |  |  |  |
| **Keywords from Thesaurus and New associated words identified through Word2Vec, if Count >110, and if not already mentioned above** | | | | | | |
|  | | | | Affliction | 242 | 0.6058 |
|  |  |  |  | Anger | 31506 | 0.4931 |
|  |  |  |  | Anxiety | 96909 | 0.6273 |
|  |  |  |  | Convulsion | 265 | 0.5125 |
|  |  |  |  | Depress | 29822 | 0.6019 |
|  |  |  |  | Depressed | 4751 | 0.5973 |
|  |  |  |  | Depression | 65223 | 0.5491 |
|  |  |  |  | Difficulty | 836 | 0.6266 |
|  |  |  |  | Distressing | 450 | 0.6071 |
|  |  |  |  | Fear | 48165 | 0.51 |
|  |  |  |  | Feeling down | 498 | 0.482 |
|  |  |  |  | Feeling tired | 198 | 0.6016 |
|  |  |  |  | Fever | 21441 | 0.5903 |
|  |  |  |  | Gasp | 810 | 0.4111 |
|  |  |  |  | Illness | 10148 | 0.6316 |
|  |  |  |  | Incision | 146 | 0.5545 |
|  |  |  |  | Injury | 11487 | 0.5158 |
|  |  |  |  | Insomnia | 12944 | 0.6033 |
|  |  |  |  | Irritable | 872 | 0.5635 |
|  |  |  |  | Irritation | 1276 | 0.5424 |
|  |  |  |  | Jerking | 174 | 0.5816 |
|  |  |  |  | Lonely | 112 | 0.5943 |
|  |  |  |  | Misery | 716 | 0.5702 |
|  |  |  |  | Nervous | 6748 | 0.579 |
|  |  |  |  | Pang | 144 | 0.5035 |
|  |  |  |  | Paranoid | 274 | 0.4672 |
|  |  |  |  | Problem | 9747 | 0.6518 |
|  |  |  |  | Puncture | 547 | 0.5408 |
|  |  |  |  | Shame | 1377 | 0.5239 |
|  |  |  |  | Sickness | 1831 | 0.5801 |
|  |  |  |  | Spasm | 4241 | 0.5341 |
|  |  |  |  | Stitch | 285 | 0.5515 |
|  |  |  |  | Stress | 16507 | 0.5832 |
|  |  |  |  | Stressed | 441 | 0.6582 |
|  |  |  |  | Suffering | 7711 | 0.6225 |
|  |  |  |  | Suicidal | 1139 | 0.7467 |
|  |  |  |  | Traction | 862 | 0.5463 |
|  |  |  |  | Trouble | 6279 | 0.6718 |
|  |  |  |  | Worry | 11468 | 0.4701 |
|  |  |  |  | Wound | 1785 | 0.5251 |
